# The Mechanical Properties of Early Aged Shotcrete under Internal Sulfate Attack

**DOI:** 10.3390/ma14133726

**Published:** 2021-07-02

**Authors:** Xiaoguang Jin, Jie He, Chao Hou, Wei Luo, Wenjun He

**Affiliations:** 1 School of Civil Engineering, Chongqing University, Chongqing 400045, China; 20201601069@cqu.edu.cn (J.H.); 20181601014@cqu.edu.cn (C.H.); 2 Key Laboratory of New Technology for Construction of China in Mountainous Area, Chongqing University, Chongqing 400045, China; 3 State Key Laboratory of Coal Mine Disaster Dynamics and Control, Chongqing University, Chongqing 400045, China; 4 Chongqing Traffic Bureau, Chongqing 404000, China; suidaol@126.com; 5 South City Power Supply Branch of State Grid Chongqing Electric Power Company, Chongqing 404100, China; caiyuzhi2850807@126.com

**Keywords:** shotcrete, early age, sulfate attack, uniaxial test, mechanical property

## Abstract

Shotcrete is the primary material for tunnel support due to its early rapid hardening characteristics. During tunnel construction in a sulfate environment, the hardening law of concrete will be affected. In this study, samples were prepared at six different curing times and immersed in four different concentrations of sulfate solutions. A uniaxial test was conducted and analyzed to investigate the effect of sulfate attack on the mechanical properties of early aged shotcrete materials. Results indicated that waterlogged shotcrete does not have apparent cracks on the outside. The stress–strain curve or ultimate compressive strength of the samples showed that the effect of sulfate on shotcrete should be differentiated into chemical and physical sulfate attacks, according to the concentration of sulfate ions. The two parameters in the equation of the hardening behaviors of sulfate attack samples, ultimate compressive strength, and time constant, are related to sulfate concentration. The crack damage stress threshold of samples demonstrates that high-concentration sulfate corrosion leads to an impact on the durability of shotcrete.

## 1. Introduction

As a significant component of an initial support structure, shotcrete is often used in tunnels and slope engineering projects. After tunnel excavation or slope construction, initial support prevents blocks from falling from walls or overhead areas. Therefore, initial support materials should provide quick strength or binding in these sections. Generally, shotcrete is a cement-based material with accelerators, and it has a quick setting time and rapid increase in strength. Hence, the properties of early aged shotcrete have attracted the attention of researchers. Galobardes et al. [1] studied the effects of the accelerator characteristics on an elastic model of shotcrete. Paglia [2] studied the effect of alkali-free and alkali-free accelerators on the early hydration rate of cement slurry. Bryne [3] indicated that the preparation method of cement pastes might significantly influence microstructure. Literature published so far shows that researchers have been more focused on the influences of different material compositions on the acceleration effect in shotcrete [4,5,6]. At the same time, engineers are more concerned about the early mechanical properties of shotcrete over time. According to the mechanical properties of accelerated concrete at different early ages, researchers could determine the parameters and the specific times for the following construction step; however, they tend to choose a more conservative approach, both in terms of design and in numerical simulations, which assumes that the initial shotcrete model is elastic and its properties are unchanged.

In addition, engineering practices sometimes have to deal with corrosive environments, e.g., the passing of the tunnels through underground areas or marine environments. In these situations, sulfate ions will affect the early age properties of shotcrete. The degradation of concrete due to sulfate ions being present in the aggregates has been widely studied in recent years [7,8]. The presence of sulfate in water or aggregates can cause the corrosion of concrete. These sulfate factors are known as internal corrosion [9,10,11].

In general, the reaction of sulfate ions with cement hydration products causes a sulfate attack. Sulfate attack on concrete is categorized as either a chemical sulfate attack (CSA) or a physical sulfate attack (PSA), depending on the sulfate exposure environment [12]. Concrete deterioration due to sulfate attack is a complex process that has been widely investigated over several decades [13,14]. Various damage mechanisms, including expansion, cracking, spalling, and loss of strength, can manifest in concrete exposed to sulfate attack. During sulfate attack, the reaction between sulfate ions and the aluminate phases of cementitious materials produce ettringite or hydrated calcium aluminum sulfate hydroxide (Ca_6_Al_2_(SO_4_)3(OH)_12_·26H_2_O) [15], which is responsible for micro-fissures and expansion. A reason for the sudden reduction in the compressive strength is the reaction of calcium silicate hydrates (C-S-H) and sulfates in producing thaumasite, which accelerates under the catalysis of ettringite [16]. In particular, the formation of ettringite and thaumasite causes the complete disintegration of the cement matrix, the loss of its main pasting phase, and consequently, making the concrete into a discrete state [17]. Unlike CSA, the main product of PSA is not the secondary product produced by the chemical reaction but the crystallization of sulfate. The appearance of crystals will lead to more significant damage due to expansion and efflorescence [18].

The accelerator is the main difference between shotcrete and ordinary concrete. The chemical compositions in the accelerator change the mechanisms of cement hydration and reduce the setting time. Therefore, the sulfate ions in the accelerator could change the property of shotcrete at an early age. Previous researchers [5,19] have documented that the sulfate used in accelerator formulation strongly affects the rate of hydration. In addition, the solubility rate of the setting regulator influences accelerator reactivity.

Although the effect of the chemical process on the accelerator has been studied, the effect of the sulfate environment on the early age properties, especially the mechanical properties of shotcrete, is not apparent. Consequently, determining the mechanical properties of early age shotcrete and the influence of sulfate ions on these mechanical properties will be helpful in engineering work. Thus, it constitutes the need to study early age behaviors of shotcrete under sulfate attack.

## 2. Materials and Methods

### 2.1. Materials

**Figure 1:**
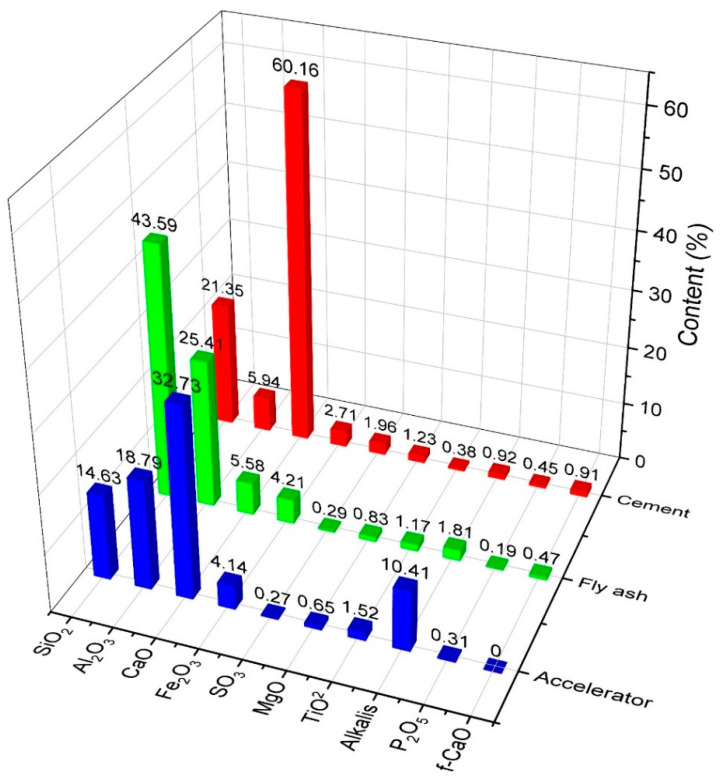
Compositions of materials used.

**Figure 2:**
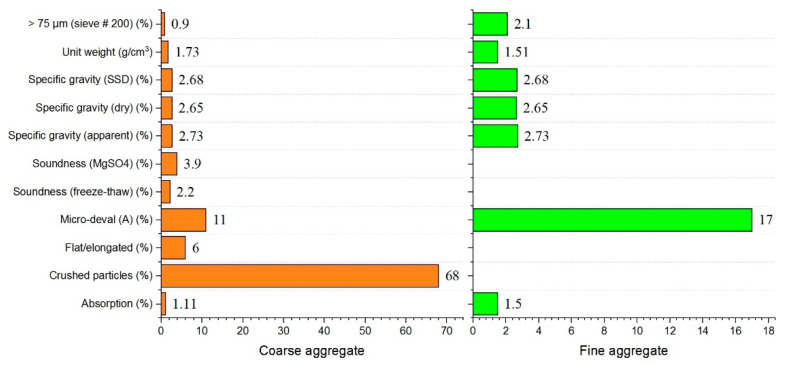
Physical and chemical properties of fine and coarse aggregates.

Ordinary Portland cement (P.O. 42.5 and C3A content of 9%) complying with ASTM Type I was used as the primary binder. Fly ash was also used to formulate the mixed proportions. The physical and chemical properties of materials used in this study are shown in Figure 1 and Figure 2 and in Table 1. All mixtures had a constant amount of binder of 464 kg/m^3^. For specific parameters, Table 2 shows the characteristics of the hardening accelerator employed during the mixing of shotcrete. Table 3 shows the compositions of the mixtures. Coarse aggregates used in this study were river gravels with a maximum size of 15 mm.

### 2.2. Sample Preparation

**Figure 3:**
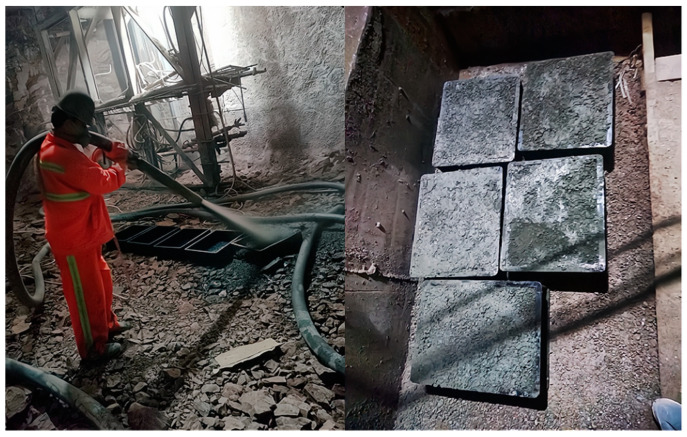
The preparation of on-site shotcrete slab.

In this experiment, the shotcrete was sprayed with a wet-mix machine at a tunnel project site (Chongqing Rail Transit Line 9, C25 shotcrete). Four groups of shotcrete were mixed with 0%, 2%, 5%, and 10% concentrations of sulfate solutions adhering to JGJ/T 372-2016 [20]. The shotcrete was sprayed on molds (450 × 350 × 120 mm^3^) (Figure 3) at the construction site and cured (23 °C (73.4 °F) and R.H. of 70%) according to the actual environmental conditions of the tunnel. After curing, the cores of size (50 mm dia and 100 mm height) were dilled out. Samples were also separated into six groups, depending on the curing time. Once each group of shotcrete samples was made, uniaxial compression tests were performed immediately. Mixes were made with different concentrations of sulfate solutions (0%, 2%, 5%, and 10%). The samples were also split into six groups according to curing time. Considering that shotcrete usually reaches its ultimate compressive strength in 7 days and begins to bear the pressure of surrounding rock within the first 12 h [20], the curing times of 4, 6, 8, 10, 12, and 24 h, respectively, were selected to measure the evolution of the compressive strengths of the mixes. A total of 90 samples were tested for the analyses.

### 2.3. Test Procedure

**Figure 4:**
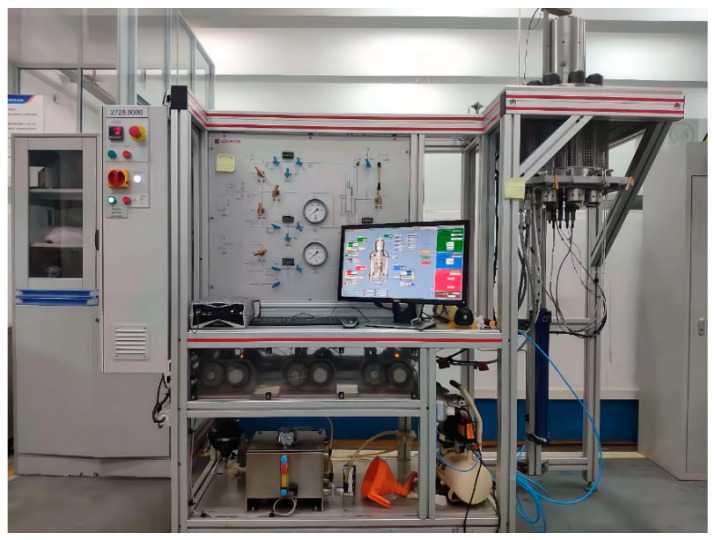
The auto-compensated and auto-equilibrated triaxle cell system.

**Figure 5:**
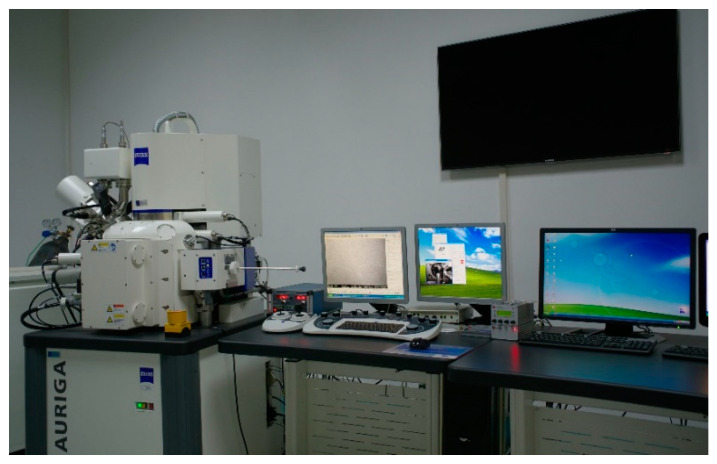
ZEISS Auriga SEM/FIB Crossbeam System.

The laboratory experiments were conducted with a TOP INDUSTER testing machine (Figure 4). The maximum axial load of this machine is 1000 kN with 60 Mpa for the maximum confining pressure. The axial and radial strains were measured using linear variable displacement transducers (LVDTs) with an axial displacement of 0–20 mm and a radial displacement of 0–5 mm. The loading rate was kept constant at 0.02 mm/min to control the strain compression limit in the test. Meanwhile, the elastic modulus, peak strain, and compressive strength of the samples were recorded. In addition, we carried out an electron microscope scanning experiment on the samples with a SEM/FIB Crossbeam System (Figure 5). The SEM (scanning electron microscope) resolution was 1.0 nm @ 15 kV, and the acceleration voltage was 0.1 kV–30 kV.

## 3. Results and Discussion

### 3.1. Failure Modes

**Figure 6:**
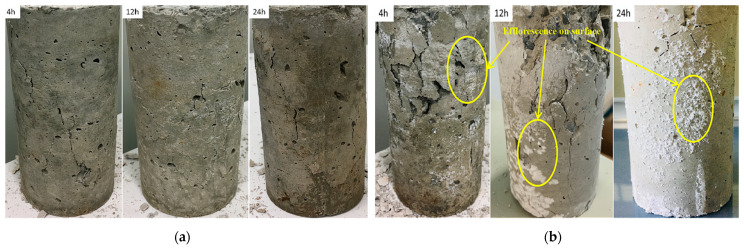
The failure of the samples under uniaxial compression: (**a**) uniaxial compression failure of samples without sulfate solution; (**b**) uniaxial compression failure of samples with 10% concentration sulfate solution.

Figure 6 shows the failure morphologies of the samples. From Figure 6a, the fracture surface of the specimen does not present common failure patterns in the samples. For example, no vertical rupture angle after failure was observed in the samples. Note that this phenomenon appeared regardless of the presence of sulfate, which means that this damage is a characteristic of early age shotcrete. From this failure mode, it can be inferred that the material had prominent plastic characteristics. Therefore, in this test, the failure could only be judged by the amount of deformation of the sample. Group D (Figure 6b), which contained samples mixed with high-concentration sulfate solution, showed different damage characteristics from group A, which was mixed with water. Then the samples were left to rest for a while; Subsequently, the samples’ surface from group D showed visible peeling and efflorescence formed on the surface; The appearance of this efflorescence phenomenon indicated that the shotcrete samples were affected by the PSA [21].

**Figure 7:**
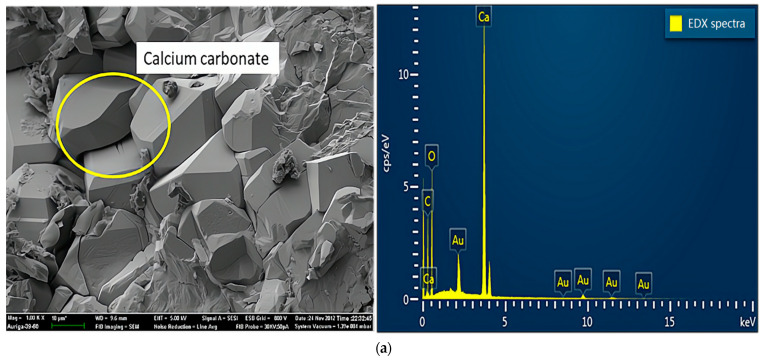

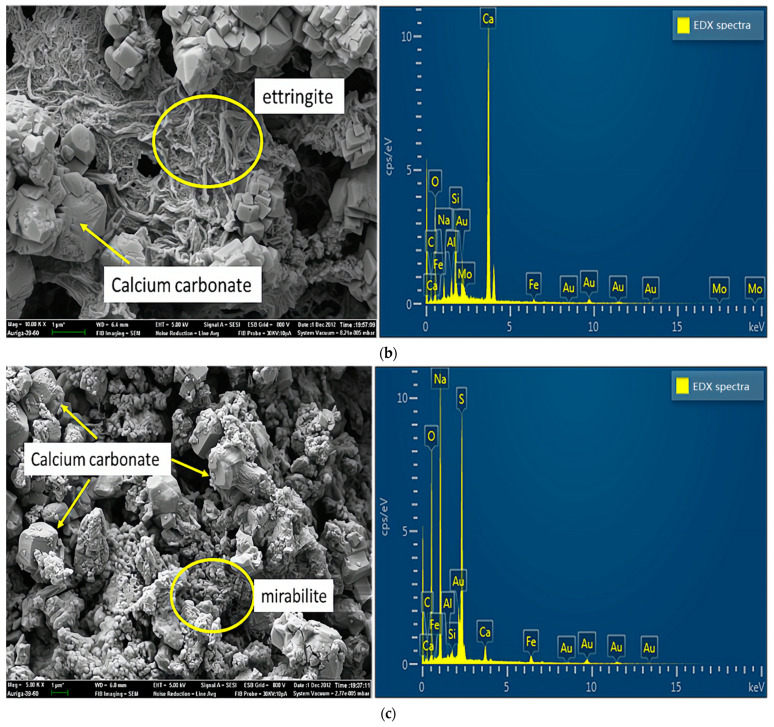
SEM and EDX spectra of samples damaged by uniaxial compression: (**a**) sample without sulfate solution; (**b**) sample with sulfate attack at 2% concentration; (**c**) samples with sulfate attack at 10% concentration.

Figure 7 shows the results of SEM and energy dispersive X-ray (EDX) analyses of specimens damaged by the uniaxial compression test. Concerning the samples from group A (Figure 7a), because portlandite crystals, the main hydration products, reacted with carbon dioxide in the air to form calcium carbonate in the preparation process of the SEM experiments, the crystals visible in the SEM experiments were mainly calcium carbonate crystals. As shown in Figure 7b, samples mixed with a 2% concentration of sulfate solution generated large amounts of ettringite, a secondary product produced by multiple chemical reactions between sulfate ions and cement [22]. In addition, ettringite is considered a typical CSA product, which leads to the degradation of concrete properties [23]. Concerning the crystals on the surface, mirabilite or thenardite, or a mixture of both (Figure 7c), were observed in small debris from the failure samples of group D. The crystals were produced due to the crystallization of excess sulfate and evaporation effects on the test piece surfaces. In other words, this process around the surface involved little or no chemical reactions. Extensive crystal formation on the surface seemed to inhibit chemical reactions. In addition, the crystals caused the peeling and efflorescence phenomenon, mentioned above, on the concrete’s surface. Therefore, the crystals (mirabilite or thenardite or a mixture of both) were considered a typical PSA product.

### 3.2. Mass Variation

Using a balance with an accuracy of 0.01 g the mass of demolded samples was measured. Subsequently, the mass variation ($M_{var}$) was calculated using Equation (1):

(1) Mvar=(mt−m0)m0×100%

where $m_{0}$ is the initial mass; $m_{t}$ is the mass of the sample after being demolded. Note that the initial mass was the mass of the samples after curing for 4 h. Therefore, a different initial mass was weighed in different groups of shotcrete samples. However, this did not affect the fact that the quality change of the samples could indirectly reflect the extent of the samples’ hydration reaction. Finally, the average mass of the samples for each curing time was taken as the sample mass.

**Figure 8:**
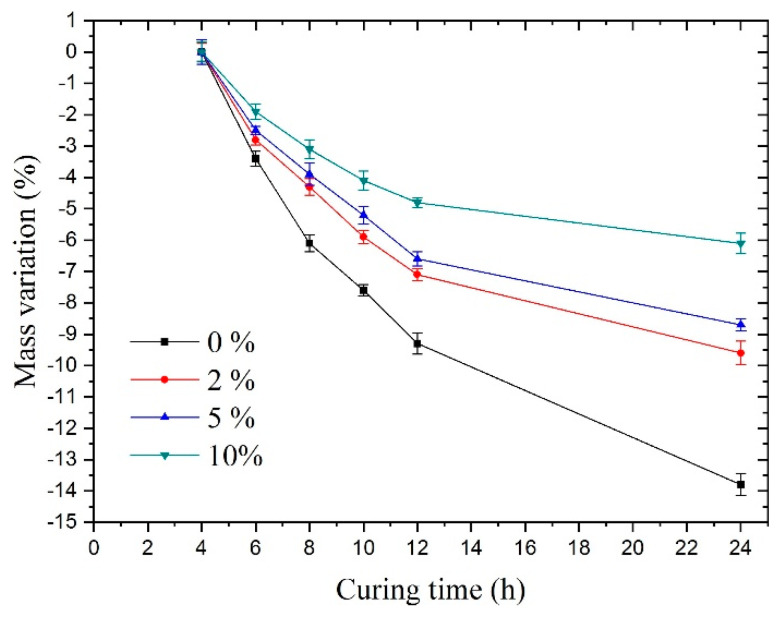
Mass variation under sulfate solutions with different concentrations.

Figure 8 shows $M_{var}$ under sulfate solutions with different concentrations. The samples always maintained a relatively fast mass loss during the 24-h curing time. In contrast, $M_{var}$ of the samples mixed with sulfate solutions exhibited a visible slowdown in this trend compared to group A’s mass variation, especially in group D (10% concentration).

For groups A, B, C, and D, the main reason for the mass loss was water evaporation. For groups B, C, and D, there were two reasons for the slowdown in the trend of mass loss rate compared with group A. One was that the production of substances increased mass, and the other was that the loss of water slowed down. More specifically, it can be explained as follows:

First, when it comes to a lower reduction in mass, according to the results of the electron microscopy experiments above, one determinable reason was that the hydration reaction of sulfate led to the production of new substances, which increased the mass. More particularly, the sulfate ions reacted with the hydrated cement paste; subsequently, its product, gypsum, could react with the cement hydration products, i.e., C3A (tricalcium aluminate), C-A-H (hydrated calcium aluminate), or monosulfate to generate ettringite, which accelerated the formation of Na_2_SO_4_ crystals. The SEM results could demonstrate this in group D, which showed a large number of crystals. Thus, a large amount of bound water was absorbed in the sodium sulfate crystals. These factors could increase the mass of the concrete.

Finally, regarding the slow loss of water, one reason for this was that the crystals absorbed a large amount of bound water and the content of free water was reduced. The other reason was that the formation of sodium sulfate crystals or ettringite changed the pore structure, making the evaporation of water slower.

As can be seen from the mass change of the samples, the sodium sulfate crystals present in the samples meant that there was interference in the concrete hydration reaction, which was consistent with the results found by Ouyang [24].

### 3.3. Stress–Strain Response

**Figure 9:**
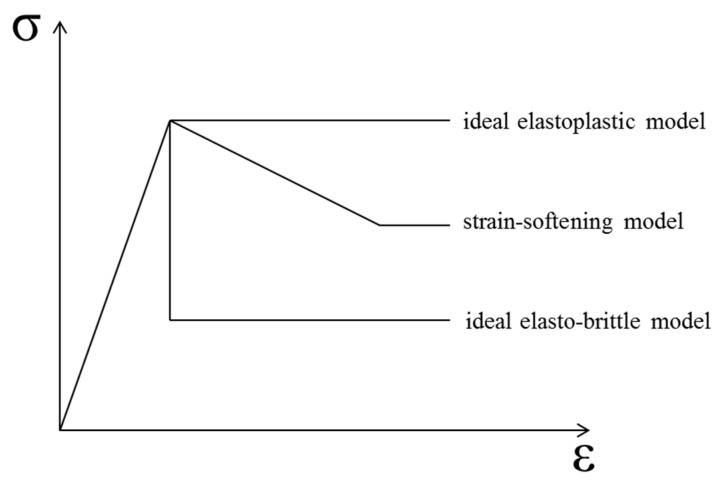
Three typical simplified post-peak curves of rock material.

The whole stress–strain curve could be obtained using the uniaxial compression test, based on the strain-controlled compression limit. Figure 9 illustrates three typical simplified post-peak curves of rock, characterized by full stress–strain curves. Since concrete is often used as a substitute material for rock in research, the rock analysis method was used to analyze the concrete material in this study. The constitutive models of the materials were separated into ideal elastoplastic, strain-softening, and ideal elasto-brittle models [25].

**Figure 10:**
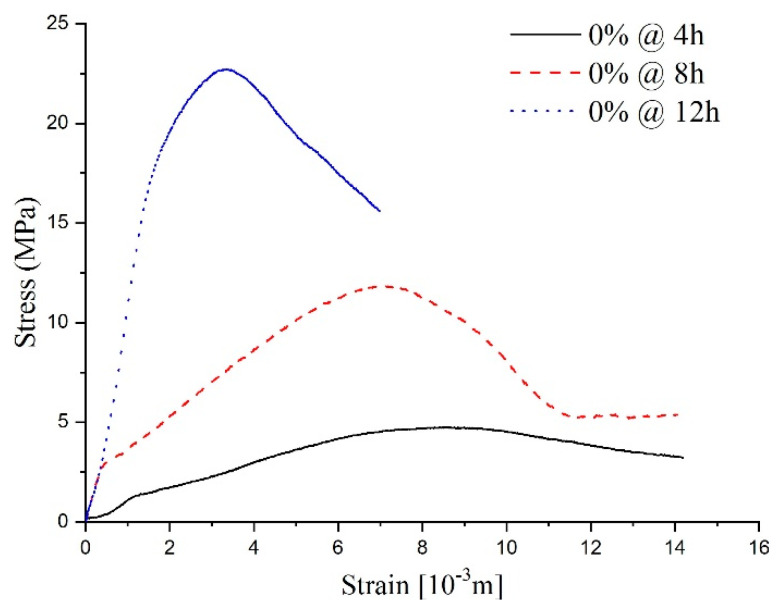
The stress–strain curve for the samples without sulfate attack.

As shown in Figure 10, the stress–strain curves at different ages of shotcrete samples are also visible differently. The stress–strain curve of the sample with a 12-h curing age is more like the typical concrete stress–strain curve, which included three typical stages. The initial stage (elastic stage), secondary stage (plastic stage), and the interior crack began to generate in the mortar during the secondary stage. Subsequently, a visible crack formed in the third stage. To facilitate comparison with specimens of other ages, a fourth stage, called the post-peak stage, was studied.

According to the fourth stage’s stress–strain curve analysis, samples with a 12-h curing age conformed to the ideal elasto-brittle model. After the sample deformation reached the peak point, the sample’s strength experienced a sudden drop to the residual value [25]. For the samples with an 8-h curing age, the curves’ first three stages were the same as the typical stress–strain curve; however, the fourth stage revealed some characteristics of the strain-softening model, one of which being that strength degrades with an increase in the main plastic strain in the post-peak stage. Concerning the shorter sample ages, like the 4 h samples, due to the low completion degree of the hydration process, the dispersion of the stress–strain curve reflects the heterogeneity of the specimen. Thus, the stress–strain curve had some erratic disturbances in the first three stages compared with the typical stress–strain curve. Nevertheless, a conspicuous strength peak could be observed, and the post-peak strength changed slowly as the strain increased. This is to say, that the characteristics in the fourth stage conform to a relatively ideal elastic-plastic model.

The stress–strain curves of specimens at different curing ages show that the fundamental properties of shotcrete change from plasticity and ductility to brittleness with age growth. The stress–strain curve’s change process can describe the hardening process of shotcrete with the hydration reaction. To be more specific, as the hydration reaction goes on, the strength increases and the fluidity decreases. Meanwhile, more and more pores become stable structures. For these reasons, under a load, deviatoric stress will be generated inside a sample, and the increase in strength makes the specimen stronger for resisting deformation caused by an external force. However, the deviatoric stress inside the specimen accelerates the collapse process of the pores and finally leads to a large amount of volume contraction [26].

**Figure 11:**
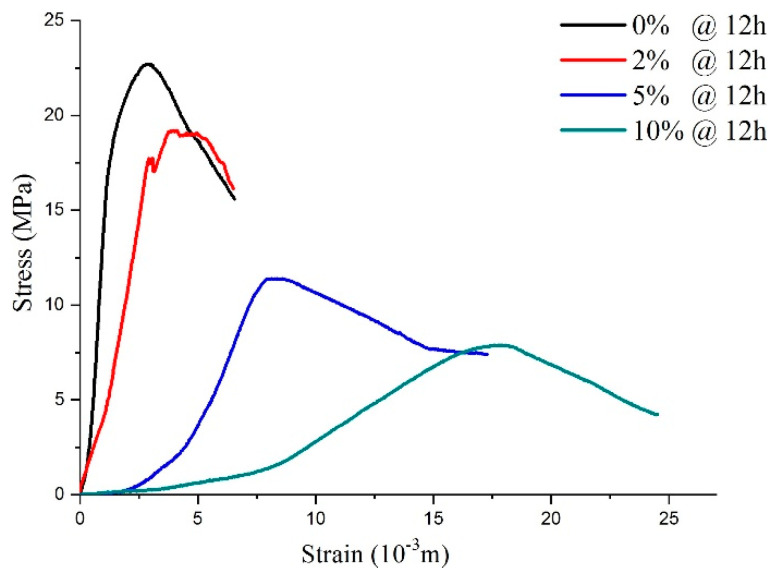
The stress–strain curve for the samples with 12-h curing age under physical sulfate attack with different concentrations.

As shown in Figure 11, sulfate attack of different concentrations on the shotcrete samples directly influences stress–strain response. After being attacked by 2% sulfate solution, although the peak strength was reduced, the stress–strain curve was similar to the curves of the water solution, which presented elastic-brittle characteristics. As the sulfate concentration increased to 5–10%, which meant a PSA on the specimens, the shape characteristic of the specimens’ stress–strain curves changed the strain-softening model. It can be inferred that the high-concentration sulfate solution added to the samples destroyed the integrity of the specimen during the hydration reaction process. This conclusion is consistent with the reasons for mass variations mentioned above.

According to the above analysis, it can be seen that with curing age growth, the post-peak mechanical behavior of the specimens changed from the ideal elastoplasticity model, the elastoplastic softening model, to the elasto-brittle model. In particular, when the curing time of all samples was 12 h (Figure 11), the CSA (concentration < 5%) acting on the concrete samples only influenced the peak strength, while the PSA (concentration ≥ 5%) acting on the samples led to a peak strength reduction and changed the shape of the stress–strain curves.

#### 3.3.1. Compressive Strength

**Figure 12:**
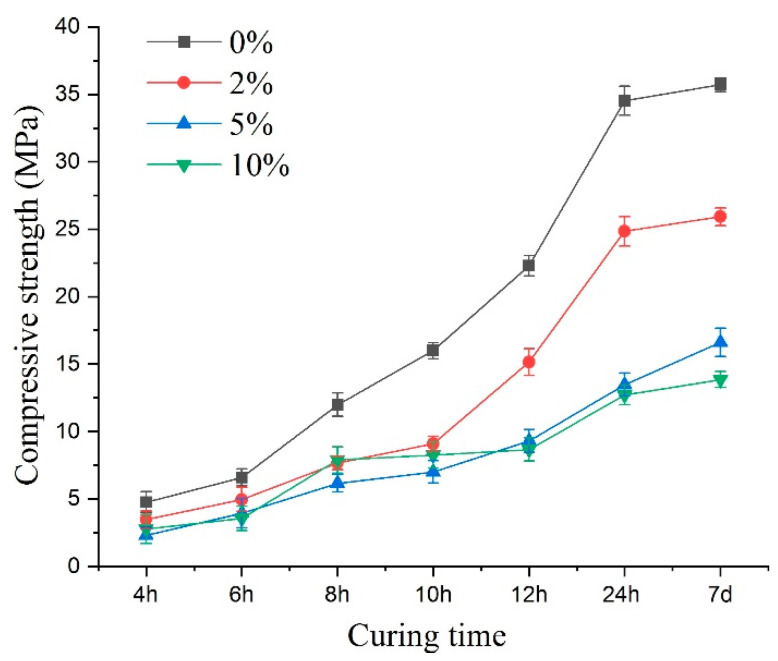
The peak compressive strength of the specimens under sulfate attack with different concentrations.

Figure 12 illustrates the compressive strength variations. Under sulfate attack, the compressive strength value of early age shotcrete samples varied with the change of sulfate solution concentration. Up to 24 h of curing, the concentration increase of the sulfate solution led to the compressive strength value showing a pronounced decrease, and the most significant decline in specimen strength reached 63.1%. Compared with the 24-h curing age samples, changing the concentration of the sulfate solution did not significantly reduce the compressive strength for the earlier age samples. More specifically, when compared with the control specimen, the compressive strength of curing age from 4 to 24 h specimens reduced by 33%, 21%, 20%, 23%, 38%, 46% for 2%; 52%, 40%, 49%, 56%, 58%, 61% for 5%; and 41%, 46%, 34%, 48%, 61%, 63% for 10% sulfate concentrations, respectively. For different concentrations of sulfate solutions, the percentage decrease in strength showed a slowing down in the process at first and then an increase. This indicated that sulfate erosion not only caused the decrease of shotcrete’s compressive strength but also had positive factors in enhancing the compressive strength of shotcrete. The variations in compressive strength were the result of the interaction of many factors. Compared to the 10% sulfate solution, during the 4–10-h curing ages, the compressive strength of shotcrete was lower when the sulfate concentration was 5%. This may be because PSA was more intense at a higher concentration in the early curing times, and more mirabilite crystals were produced, which compacted some pore structures and slowed the reduction of compressive strength.

When the sulfate concentration increased to more than 5%, the compressive strength value did not decrease significantly with the concentration increase, and the corrosion type of the samples changed from CSA to PSA, which could be demonstrated by the crystals in the picture in Figure 7c. The increase in the sulfate concentration did not result in a linear reduction in the samples’ strength.

In the early stage of PSA, sodium sulfate solution entered the shotcrete specimen and subsequently precipitated crystals in the pre-existing pores, which resulted in a slight increase in mass. As the attack progressed, the pore crystals gradually increased, and the crystals grew gradually towards the pore wall. Meanwhile, due to the crystallization pressure, cracks appeared near the pore wall. As erosion continued, larger cracks developed and coalesced, leading to the concrete’s deconstruction and the decrease in strength. By comparing the 7-day strength of specimens with different concentrations (Figure 12), i.e., the ultimate compressive strength after curing, it could be seen that the physical erosion of sulfate had a significant influence on the ultimate compressive strength of shotcrete. This result showed that the decreasing compressive strength of the concrete specimens caused by PSA was more remarkable than those of CSA.

The compressive strength of samples with different curing times can also determine the hardening behavior of the materials. In regards to the hardening law of the concrete, which links the ultimate compressive strength with time, previous researchers came to the following equations [27]:

(2) $$\sigma_{cls.t}=\sigma_{cls.0}\cdot \left(1-e^{-\beta t}\right)$$

(3) $$E_{cls.t}=E_{cls.0}\cdot \left(1-e^{-\beta t}\right)$$

where $\sigma_{cls.0}$ is the 28-day ultimate compressive strength. $E_{cls.0}$ is the 28-day elastic modulus. β is time constant [$t^{-1}$]. $\sigma_{cls.t}$ is the compressive strength at time t. $E_{cls.t}$ is the elastic modulus at time t.

**Figure 13:**
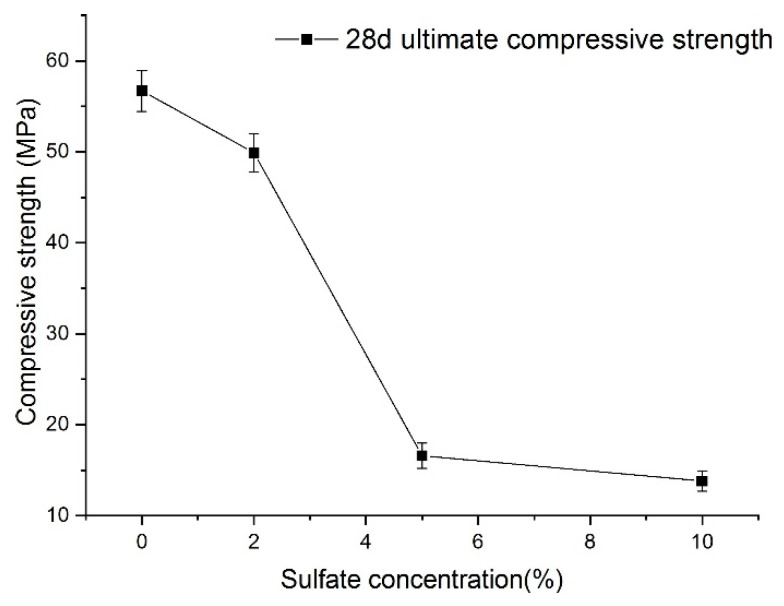
Relationship between sulfate concentration and the final strength of samples.

Nevertheless, Figure 13 shows the relationship between sulfate concentration and the 28-day (according to the specifications) ultimate compressive strength. A visible characteristic of the 28-day ultimate compressive strength value of shotcrete samples is that it decreases as sulfate concentration increases. With the increase in sulfate concentration, the decrease in the 28-day ultimate strength showed accelerating and then slowing down. Because of this, the 28-day ultimate compressive strength of shotcrete material cannot directly reflect the function law for the sulfate concentration. Even so, it can be seen that when the concentration of sulfate is large, the strength of the samples decreases significantly.

**Figure 14:**
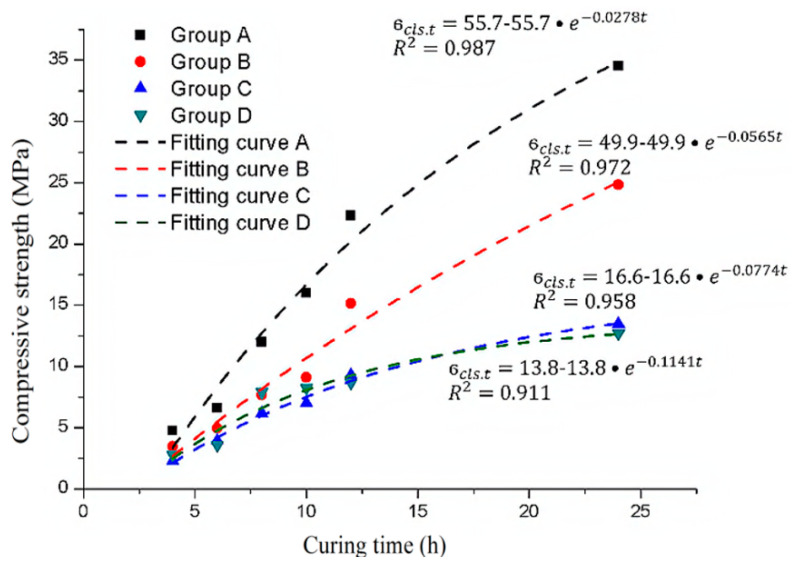
The experimental data and exponential function fitting curve of the ultimate compressive strength of the samples with different sulfate solution concentrations.

Hence, for shotcrete, the 7-day compressive strength is generally taken as the ultimate compressive strength [20]. Therefore, the 28-day compressive strength in the concrete hardening formula is replaced by the 7-day strength of shotcrete to obtain the shotcrete strength hardening formula. This formula is used to fit the experimental data, and the results are shown in Figure 14.

In Figure 14, according to the correlation coefficients (R^2^), the exponential function derived from the concrete material can still better describe the hardening process of shotcrete. The main difference between the samples mixed with different sulfate solution concentrations is the final strength and the constant time parameter. Therefore, it can be considered that both the ultimate compressive strength of the samples and the time constant are related to the sulfate concentration.

**Figure 15:**
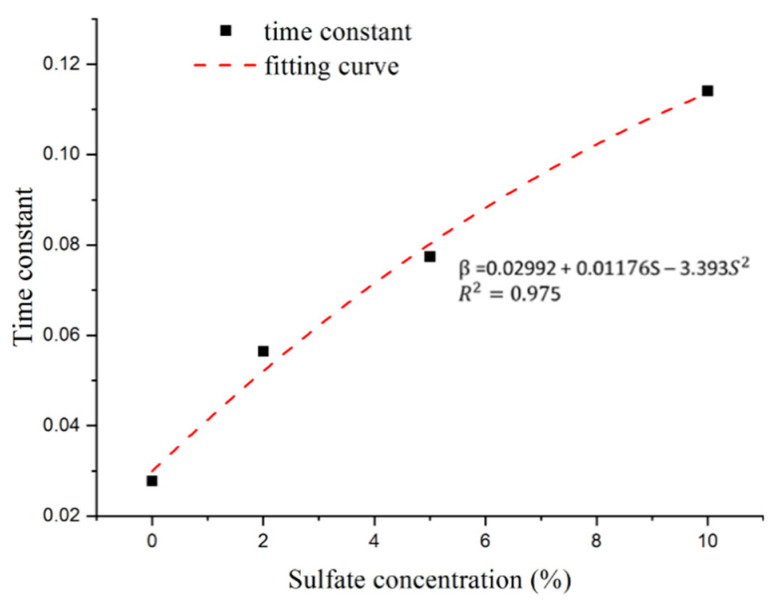
Relationship between sulfate concentration and time constant.

As shown in Figure 15, the time constant increases with increasing sulfate concentration. The relationship between the time constant and sulfate concentration shows a clear functional relationship. Thus, the appropriate formulas of the time constant can be expressed through polynomial forms with a correlation coefficient (R^2^) greater than 0.9.

#### 3.3.2. Crack Damage Stress Threshold

Martin and Chandler [28] first proposed the crack damage stress ($\sigma_{cd}$) threshold as the long-term strength of a specimen. In other words, regardless of whether the stress reaches the failure stress of the specimen or not, when the stress remains constant at $\sigma_{cd}$, the sample will eventually be destroyed. In particular, the lower limit is usually used to analyze spalling in tunnels [29]. Considering the similarity between rock mass material and concrete material, which is often used as an alternative in rock experiments, the crack damage stress ($\sigma_{cd}$) threshold value was used to research sulfate influence on shotcrete samples in this study.

Meanwhile, the influence of the sulfate factor on $\sigma_{cd}$ will directly affect the durability of shotcrete. The crack damage stress $\sigma_{cd}$ is from the stage of the stress–strain curve. It is generally accepted that the compression stress–strain curve can be divided into different stages, characterized by several stress thresholds, including crack closure stress $\sigma_{cc}$, crack initiation stress $\sigma_{ci}$, and crack damage stress $\sigma_{cd}$ [26]. Once the stress reaches $\sigma_{cd}$, the volume will reach the maximum and the material will enter an accelerated crack stage. The crack propagation is mainly due to the generation of new cracks, old crack growth, and the slip between crack surfaces. The stress corresponding to the maximum volumetric strain point in the curve is determined to be $\sigma_{cd}$. Usually, the method has high precision and low subjectivity. The volumetric strain under triaxial compression is:

(4) $$\varepsilon_{v}=\varepsilon_{1}+2\varepsilon_{3}$$

where $\varepsilon_{v}$ is the volume strain. $\varepsilon_{1}$ and $\varepsilon_{3}$ are the axial and lateral strains, respectively. Martin [26] divided the deviatoric stress curve corresponding to the axial strain into five stages and took the stress threshold value of the fourth stage (the crack instability development stage) as crack damage stress $\sigma_{cd}$. In the uniaxial compressive test, the third principle ($\sigma_{3}$) stress always equals zero. Therefore, the deviatoric stress equals to first principal stress ($\sigma_{1}$).

**Figure 16:**
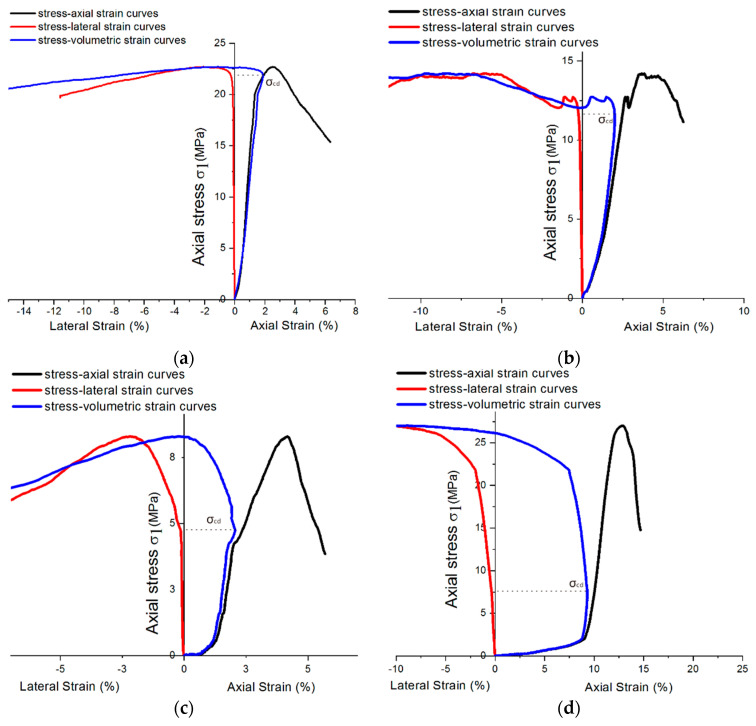
Stress–strain diagram showing $\sigma_{cd}$ of 12-h curing age samples mixing with different sulfate concentrations: (**a**) 0% concentration; (**b**) 2% concentration; (**c**) 5% concentration; and (**d**) 10% concentration.

Due to the influence of the hardening accelerator, the shotcrete used in the tunnel was required to bear the weight as a tunnel support structure after curing for 12 h. Therefore, this paper uses the shotcrete group with a 12-h curing time to analyze $\sigma_{cd}$. For the sake of better describing the relationship between volumetric strain and crack propagation under uniaxial loading conditions, $\sigma_{cd}$ of samples under sulfate attack with different concentrations are compared, as shown in Figure 16. Although the compression stress–strain curve cannot show the shotcrete material’s various stages, $\sigma_{cd}$ can still be located by the turning point of the volume curve, which is also the maximum point of volumetric strain [26].

Furthermore, the curves show that the sample expanded rapidly to the failure stage when the volume strain shrank to a certain extent. It can be inferred that there were many microcracks in the concrete with the accelerator, which produced a more protracted period of volume strain contraction. As the sulfate solution concentration reaches 5%, the specimens’ lateral strains develop earlier, which leads to volume strain dilation earlier. According to Section 3.1, the volume strain expansion in high-concentration sulfate solution may be attributed to the crystallization expansion caused by PSA. There is a synchronous trend between $\sigma_{cd}$ in Figure 16 and $\sigma_{c}$ (uniaxial compression strength) in Figure 14. As $\sigma_{c}$ decreases, $\sigma_{cd}$ also tends to decrease. Because of this, the ratio $\sigma_{cd}/\sigma_{c}$ could be used as a quantitative index for comparison and basic inherent property of rock, which could be used to predict the failure process in rock engineering [25]. This index could also be used to analyze the shotcrete samples in this study.

**Figure 17:**
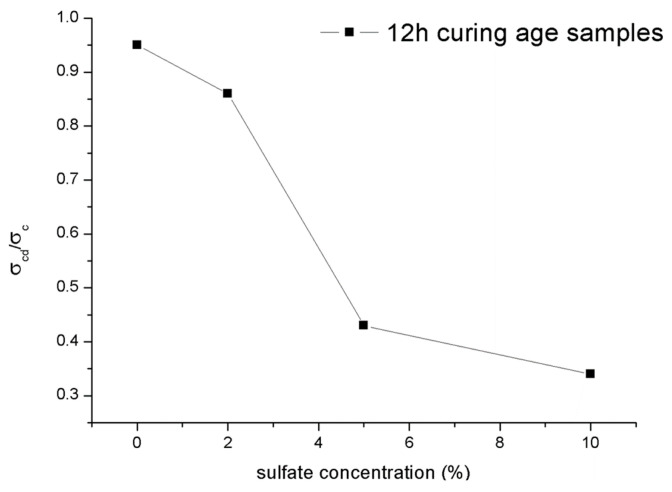
Ratio $\sigma_{cd}/\sigma_{c}$ for samples under different concentrations of sulfate attack.

Figure 17 shows the ratio $\sigma_{cd}/\sigma_{c}$ for the 12-h curing time samples with different concentrations of sulfate attack. The attacks caused by sulfate solutions in shotcrete would lead to the ratio $\sigma_{cd}/\sigma_{c}$ decreasing. Like the character of the ultimate strength shown in Section 3.3.1, the ratio $\sigma_{cd}/\sigma_{c}$ of the samples also decreased significantly as the concentration of sulfate solutions increased from 2% to 5%. In general, the normalized data, particularly for the material of rocks, are mainly distributed in a relatively narrow range between 0.6 and 0.9 [28], while the ratio of $\sigma_{cd}/\sigma_{c}$ of the samples mixed with water and 2% sulfate solution also conform to this regularity. In contrast, the ratio $\sigma_{cd}/\sigma_{c}$ of samples under PSA would be less than 0.6. It can be inferred that once the concentration of sulfate solution exceeds a specific limit, PSA was entered. Moreover, this similar law between the ultimate strength and the crack damage stress may mean that the change in the crack damage stress is the cause of the change in the ultimate strength. Mixing with excessive sulfate solution concentration may mean earlier microfracture generation and the rapid growth of cracks in the shotcrete. Subsequently, the samples show a decrease in volumetric strain. In addition, this microcrack generation and crack growth will decrease the setting compressive strength.

## 4. Conclusions

The present study focused on internal sulfate attack on early age shotcrete. The shotcrete samples mixed with sulfate solutions (instead of water) were produced by spraying large plates at a construction site, then testing the early performance of concrete through uniaxial compression. The main conclusions derived from this work are presented:

1. Regardless of sulfate factors, the failure characteristics of shotcrete did not show objective cracks on the outside. The damaged specimens showed physical sulfate attack damage characteristics, and the efflorescence phenomenon only under high concentrations of sulfate. SEM and energy dispersive X-ray (EDX) analyses of specimens showed that lower sodium sulfate concentration, such as 2%, led to a great deal of ettringite formation. Samples mixed with 5% and 10% concentration sulfate solutions had crystals that appeared on the sample surfaces.
2. The curing time significantly affects the stress–strain relationship of early age concrete with a hardening accelerator. Regardless of the stress–strain curve or the ultimate compressive strength law of the shotcrete material, the properties of shotcrete will deteriorate due to the presence of sulfate. Simultaneously, the effect of sulfate on shotcrete should also be differentiated into chemical sulfate attack and physical sulfate attack, according to sulfate concentration.
3. The concrete hardening behavior equation expressed by the elastic modulus and the compressive strength can describe the hardening behavior of shotcrete by modification. The fitting result could be accepted according to the correlation coefficients. Meanwhile, the two parameters in the equation of the sulfate attack samples, the ultimate compressive strength and the constant time are related to sulfate concentration.
4. In a stress–strain curve, when the volume strain reaches a certain extent, the samples will enter the rapid expansion stage. As the sulfate solution concentration increases from 2% to 5%, the specimen’s lateral strain develops much earlier, which leads to earlier volume strain dilation. Moreover, as the concentration of sulfate solution increases from 2% to 10%, the ratio $\sigma_{cd}/\sigma_{c}$ of samples decreased significantly, which could illustrate the similar trend of the ultimate compressive strength of samples. The ratio $\sigma_{cd}/\sigma_{c}$ of samples under a high concentration, sulfate attack is less than 0.6, which less than the index of rock material. In other words, the sulfate attack caused by the solution with a high concentration leads to an impact on the shotcrete’s durability.
5. In practical engineering, we suggest that water quality be tested, and the aggregates should be strictly selected. Sulfate ions in water are not up to standards, which may cause the strength of shotcrete to decline sharply. Moreover, the aggregates can precipitate sulfate ions and increase the sulfate concentration of the overall concrete material. In this case, it may also cause local performance degradation caused by the local sulfate content being too high and then affect the overall performance of concrete.

## Figures and Tables

**Table 1:** Properties of materials used.

Physical Properties	Cement	Fly Ash
Density (g/cm^3^)	3.12	2.11
Blaine fineness (cm^2^/g)	3345	4042
Loss of ignition (%)	2.25	3.1

**Table 2:** Physical properties of hardening accelerator.

Main Component	Type	Density (g/cm^3^)	Usage (Cement × %, Mass Ratio)
Calcium aluminate	Powder	2.15	1–5

**Table 3:** Mixture proportion of shotcrete.

S No	Cement	Coarse Aggregate	Fine Aggregates	Superplasticizer	Accelerator	Water	Fly Ash	Sulfate Ion Concentrations (%)	w/c
	(kg)								
A (Control)	486	844	813	4.86	12.4	235	45	0	0.5
B	486	844	813	4.86	12.4	235	45	2	0.5
C	486	844	813	4.86	12.4	235	45	5	0.5
D	486	844	813	4.86	12.4	235	45	10	0.5

## Data Availability

Partial or full data will be available on request from the corresponding author.
